# Upon the Pleasures and Advantages of a Knowledge of the Natural Sciences

**Published:** 1854-07

**Authors:** 


*Upon the Pleasures and Advantages of a Knowledge of the Natural Sciences.*

It is this training of the mind in correct methods of observation that gives the Natural History sciences so much value as instruments of preparation in professional education. Not unfrequently do we hear the short-sighted and narrow-minded ask, what is the use of zoology, or botany, or geology to the physician and surgeon? What have they to do with beasts, or plants, or stones? Is not their work among men, healing the sick? Of what use, save as remedies, can the creeping things, or the grass that grows upon the earth, or the minerals in the rock, be to the practisers of medicine? Vain and stupid questions all—yet they are sometimes put by persons who profess to promote the spread of education. We hear the same outcry on the literary side of teaching. What is the use of Greek and Latin? Can Greek make a man successful in bargaining, or Latin add to his riches? Why teach philosophy—the world is not for philosophers? What is rhetoric to the farmer? Who has not heard these questions asked over and over again? Yet always by professing advocates of education—practical education. They want something, but the best of them mistake the end for the means. The best want knowledge, but have not learnt that the mind must be trained ere it is prepared to gather and digest knowledge. They want science, but science turns mouldy and unwholesome in an unprepared mind. They forget, or do not know, that education consists chiefly in training, not in informing. That is instruction. At the same time, without a due mixture of instruction, education becomes insipid and distasteful to boyhood and youth. The older the pupil the more instruction must be mingled with the teaching. And when we are professionally educating young men, then the more science we can instil through our educatory lessons the better for them. Were the sciences so infused, to be entirely professional, we should warp and contract the minds. The tonic would be too strong—would not invigorate, but corrugate. We must counteract the natural tendency of purely professional studies—the tendency to limit the range of mental vision. We can do this most beneficially through the collateral sciences, which are sufficiently allied to the professional ones to prevent an undue dissipation of the student's thoughts, and at the same time are sufficiently different to give them a wider sphere of action. It is in this point of view that we should regard the Natural History sciences as branches of medical education. For my own part, after much intercourse with medical men who had studied at many seats of professional education, some collegiate, some exclusively professional, I have no hesitation in saying that, as a rule, the former had the intellectual advantage. There are noble and notable exceptions, old and young; but the rule is true in the main. The man who had studied in a seat of learning, a college or university, has a wider range of sympathies, a more philosophical tone of mind, and a higher estimate of the objects of intellectual ambition than his fellow-practitioner, who, from his youth upwards, had concentrated his thoughts upon contractedly professional subjects of an hospital school. I will not believe that the practitioner of medicine, any more than the clergyman, or the lawyer, or the soldier, or the merchant, is wiser or better able to treat the offices of his calling, because his mind takes no note of subjects beyond the range of his professional pursuit. It is a great pleasure, both to patient and neighbor, to find in our doctor an enlightened friend—one who, whilst he does his duty ably and kindly, has a sympathy and an acquaintance with science, or literature, or art. Such men have gone forth in numbers from this University, and have done much to contribute to its fame. May there be many of them sent out for centuries to come. Of those who come here to study professionally, there are not a few who may some day find themselves isolated in distant and little-explored regions. Far away from friends and the conversation of intellectual companions, any pursuit which can engage and occupy the mind, and above all satisfy its thirst for truth by draughts from the pure and refreshing fountains of nature—any such pursuit becomes a blessing, and converts the desert into a paradise, one often filled with creatures yet to be named. How delightful does it then become to be able to recall the lessons of our student days, and casting away regret and languor, invigorate our minds by the practice of healthy intellectual exercise. Through no branch of knowledge can this be attained more easily and more excellently than through natural history.

In all directions around us the subjects for our study abound. We require few instruments, and those seldom of a costly character. A few books will serve as notes upon the great volume of nature, whose pages, after all, are the best manuals as well as the fullest cyclopædias. The cultivation of good, strong, straightforward common-sense—that sort which generates a logical frame of mind—is the best preparation, and one which is neither rare nor technical. Faith in science—a just but not blind trust in authority—a firm determination to be cautious and careful, and a resolute and patient spirit of perseverance—these are the qualities that will ensure success for our endeavors. Through them we may advance to discovery, if we have the will, power, and opportunity of doing original work. If our aims be less ambitious, we may, by turning them to good account in our study of animated and inanimated nature, exercise our minds in habits of minute and accurate observation, of systematic comparison, and of philosophical generalization. We shall find ourselves in the end to have gone through an education of a kind that can qualify us for the strife of life and the ways of men, far removed as they may seem from the subjects of our studies, and as surely prepared for scientific investigation of no light order. It matters little how few the facts studied may be, provided they were sufficient. It matters less whether the knowledge so acquired is useful, in the vulgar sense of the term, or not. It matters much, very much, that we have gained in mental training, and that, through pursuing the line of study just indicated, we have become prepared for understanding the facts and phenomena of the world about us, and acquired a capacity to comprehend the laws and the three great kingdoms of nature.—*Inaugural Lecture, by Prof. Edward Forbes, of the University of Edinburgh.*

*Edinburgh Monthly Journal of Medical Science.*

